# LPCAT1 promotes gefitinib resistance via upregulation of the EGFR/PI3K/AKT signaling pathway in lung adenocarcinoma

**DOI:** 10.7150/jca.66126

**Published:** 2022-03-21

**Authors:** Jianghua Ding, Xinjing Ding, Zhaohui Leng

**Affiliations:** 1 Department of Hematology & Oncology, The Affiliated Hospital of Jiujiang University, Jiujiang, Jiangxi Province, China.; 2 Department of Undergraduate, The Medical College of Nanchang University, Nanchang, Jiangxi Province, China.

**Keywords:** LPACTA1, gefitinib resistance, EGFR/PI3K/AKT signaling, lung adenocarcinoma

## Abstract

Currently, the mechanisms of epidermal growth factor receptor (EGFR) tyrosine kinase inhibitor (TKI) resistance have been a focus of clinical research. Despite that most of the mechanisms of acquired EGFR TKI resistance have been revealed, about 30% of non-small-cell lung cancer (NSCLC) cases have not been fully elucidated, especially for lung adenocarcinoma (LUAD). Recently, LPCAT1, an important enzyme of phospholipid metabolism, has been found to bridge the gap between the oncogene and metabolic reprogramming. In NSCLC, LPCAT1 has been shown to participate in progression and metastasis. However, little is known about the role of LPCAT1 in acquired EGFR TKI resistance. In this study, elevated LPCAT1 expressions were observed in an EGFR TKI-resistant cell line (PC-9R) relative to a corresponding EGFR TKI-sensitive cell line (PC-9). *In vivo* and *in vitro* gene functional studies showed that LPCAT1 contributed to the pathogenesis of gefitinib resistance in LUAD, where an LPCAT1-EGFR positive feedback loop formed and then regulated its downstream signaling molecules of the EGFR/PI3K/AKT signaling pathway. The results provided novel insights into the acquired resistance mechanism of EGFR TKI from the perspective of phospholipid metabolism. These findings suggest LPCAT1 may serve as a potential therapeutic target for patients with EGFR TKI-resistant NSCLC.

## Introduction

Lung cancer is the leading cause of cancer mortality in China, with 714,699 deaths from the disease recorded in 2020 1. Non-small-cell lung cancer (NSCLC) constitutes approximately 85% of all lung cancers, and lung adenocarcinoma (LUAD) is the most common histological subtype of NSCLC, accounting for 80% of cases 2. The prognosis of advanced LUAD patients has been revolutionized due to the continual advent of novel target molecular therapies, in particular for patients harboring epidermal growth factor receptor (EGFR) gene mutations. Thus, first-line EGFR tyrosine kinase inhibitor (TKI) monotherapies, such as gefitinib, erlotinib, and osimertinib, are currently recommended as standard treatment. To date, the longest reported medial progression-free survival is 18.9 months for osimertinib 3. Meanwhile, owing to its low price, gefitinib has become the most widely used drug in China by advanced NSCLC patients carrying EGFR-positive mutations. Unfortunately, almost all of these patients eventually develop acquired resistance to EGFR TKI, which directly affects their clinical prognosis and overall survival.

Besides the classical EGFR-T790M mutation and transformation to a small-cell lung cancer (SCLC) phenotype, aberrant activations of bypass signaling pathways responsible for EGFR TKI resistance have been identified, such as c-MET amplification and abnormal activation of the PI3K/AKT pathway 4. In particular, EGFR/PI3K/AKT signaling is confirmed to participate in the pathogenesis of chemoresistance in NSCLC cells 5. However, the acquired mechanism of EGFR TKI resistance remains unknown for about one-third of EGFR TKI-resistant lung cancer patients. Therefore, there is an unmet clinical need to clarify new mechanisms governing EGFR TKI resistance.

As is well known, metabolic reprogramming is becoming a hotspot in the field of cancer research. Cancer cells can alter their capability to metabolize carbohydrates, lipids, and proteins in an effort to strive for cell proliferation and survival, including lung cancer cells 6-9. Recently, lysophosphatidylcholine acyltransferase 1 (LPCAT1), a lipid metabolic enzyme, was reported to be overexpressed and to function as a tumor-promoting role in several types of cancers, such as prostate cancer, gastric cancer, and liver cancer 10-12. In brain glioma cells, LPCAT1 contributes to the remodeling of membrane lipids and induces activation of the EGFR signaling pathway on the cellular membrane, which stimulates cell proliferation and further fuels the synthesis of LPCAT1 13. These results demonstrate that a positive feedback regulatory mechanism exists between LPCAT1 and the activation of EGFR signaling, which can be named as the “LPCAT1-EGFR feedback loop” 13. The above results strongly suggest a potential association between LPCAT1 and the EGFR signaling pathway in patients with NSCLC. Furthermore, LPCAT1 promotes brain metastasis of LUAD via upregulation of the PI3K/AKT/MYC pathway 14. However, the role of LPCAT1 in gefitinib-resistant NSCLC cells remains unclear.

In the present report, three cell lines (MRC-5, PC-9, and PC-9R) representing normal human embryonic lung cells, gefitinib-sensitive cells harboring an EGFR exon 19 deletion mutation, and gefitinib-resistant cells, respectively, were selected. Gefitinib was chosen as the research agent due to the wide usage of EGFR TKI in China. We first explored the baseline level of LPCAT1 among three cell lines, and our data revealed that LPCAT1 expression was significantly higher in PC-9R cells than in PC-9 or MRC-5 cells. Then, LPCAT1 knockout was performed and found to be strongly associated with restoring the sensitivity to gefitinib in the PC-9R cell line. Finally, the PI3K/AKT pathway was confirmed to positively participate in the development of LPCAT1-mediated gefitinib resistance both *in vitro* and *in vivo*.

## Materials and methods

### Cell lines, animals, and reagents

PC-9 and PC-9R cell lines were purchased from Beinabiology Co., Ltd. (Beijing, China), and the MRC-5 cell line was purchased from Procell Life Science & Technology Co., Ltd. (Wuhan, China). Five-week-old male BALB/c nude mice were obtained from Hunan SJA Laboratory Animal Co., Ltd. (Changsha, China). Gefitinib (CAS: 184475-35-2) and the inhibitor of PI3K/AKT pathway, LY294002 (CAS: 154447-36-6), were purchased from Shanghai Yuanye Bio-Technology Co., Ltd. (Shanghai, China) and MedChemExpress (Monmouth Junction, NJ, USA), respectively.

### Cell culture

PC-9, PC-9R, and MRC-5 cell lines were cultured in Roswell Park Memorial Institute 1640 medium (Gibco, Grand Island, NY, USA) containing 10% fetal bovine serum and 1% double antibiotics (Solarbio, Beijing, China) and then maintained in standard conditions (i.e., 5% CO_2_ and 95% atmosphere, 37 °C). The medium was changed every three days and cells were passaged with 0.25% trypsin for digestion when the cell density reached 80% to 90% confluence.

### Cell transfection

After the cells reached 80% confluence, the medium was exchanged for a serum-free medium. Cell transfection was performed with Lipofectamine 2000 transfection reagent (Invitrogen, Carlsbad, CA, USA) according to the instruction manual. PC-9 and PC-9R cell lines stably expressing LPCAT1-specific short hairpin RNA (shRNA) or scrambled control-shRNA were constructed using a lentiviral shRNA technique from GeneChem Company (Shanghai, China). The LPCAT1 shRNA was designed based on the small interfering RNA (siRNA) sequence (GenBank identification no.: 79888).

The primer used to obtain the overexpression sequence of LPCAT1 is as follows: F: 5'-CCCTGTGACCATGACGATGT-3', R: 5'-TGAATGCACCAGGTTTGAAGGT-3'. Meanwhile, the primer used for the overexpression negative control (NC) of LPCAT1 is as follows: F: 5'-UUCUCCGAACGUGUCACGUTT-3'; R: 5'-ACGUGACACGUUCGGAGAATT-3.

Separately, the employed LPCAT1 shRNA sequences are as follows: (1) F: 5'-GGACAG AUACUCAGAAAGATT-3', R: 5'-UCUUUCUGAGUAUCUGUCCTT-3'; (2) 5'-CCAUUG ACCAAGAGGAGAATT-3', R: 5'-UUCUCCUCUUGGUCAAUGGTT-3'; and (3) F: 5'-CUGAGGAGGAGAAGAGGAATT-3', R: 5'-UUCCUCUUCUCCUCCUCAGTT-3'. Finally, the sequence of carboxyfluorescein (FAM)-labeled NC siRNA is as follows: F: 5'-UUCUCCGAACGUGUCACGUTT-3'; R: 5'-ACGUGACACGUUCGGAGAATT-3'. All primer pairs were synthesized by Beyotime (Shanghai, China).

### Cell counting kit 8 (CCK-8) assay

A cell counting kit 8 (CCK-8) assay was conducted using a CCK-8 kit from KeyGEN Biotechnology (Nanjing, China). The PC-9 and PC-9R cell lines were plated in 96-well plates at 1.0 × 10^4^ cells/well, and then treated with different concentrations of gefitinib** (**5 nM, 500 nM, 1 μM, 20 μM, or 40 μM**)** for 48 hours. The cells were subsequently incubated with a 10% solution of CCK-8 at 37 °C for two hours. Finally, the absorbance at 450 nm was measured using a multifunctional enzyme-linked analyzer (Bio-Tec, VA, USA). Each experiment was repeated at least three times.

### Flow cytometry of cell apoptosis

A total of 1 × 10^6^ cells from various groups were collected, washed with phosphate-buffered saline (PBS) and suspended in 500 μL of binding buffer. Then, the cells were stained with an annexin V-fluorescein isothiocyanate/propidium iodide apoptosis detection kit (BD Biosciences, San Diego, CA, USA) at room temperature for 10 minutes according to the manufacturer's instructions. Cell apoptosis was analyzed by flow cytometry.

### Quantitative reverse transcription-PCR (qRT-PCR) assay

Quantitative real-time transcription-PCR (qRT-PCR) was used to detect the messenger RNA (mRNA) expression of the *LPCAT1* gene. Total RNA was extracted using the Ultrapure RNA kit (CoWin Biosciences, Beijing, China); then, reverse transcription was conducted using the HiScript® II Q RT SuperMix for qPCR (Vazyme, Nanjing, China). qRT-PCR was performed with 2× SYBR Green PCR Master Mix (Lifeint, Xiamen, China) on a real-time fluorescent quantitative PCR meter (CFX Connect; Bio-Rad Laboratories, Hercules, CA, USA). The β-actin gene was used as an internal reference gene, and the data were analyzed using the 2^-ΔΔct^ method. PCR primers were ordered from Beyotime (Shanghai, China). All experiments were conducted according to the manufacturer's protocols. Experiments were performed at least in triplicate.

The PCR primer sequences used are as follows: LPCAT1 (F) 5'-CCCTGTGACCATGACGATGT-3, (R) 5'-TGAATGCACCAGGTTTGAAGGT-3' and β-actin (F) 5'-TGGCACCCAGCACAATGAA-3', (R) 5'-CTAAGTCATAGTCCGCCTAGAAGCA-3'.

### Western blotting assay

The cells collected from various groups were lysed in radioimmunoprecipitation assay lysis buffer (Applygen, Beijing, China). Subsequently, the cells were centrifuged for 10 minutes at 12,000 rpm. Then, the supernatants were taken, and total protein was determined via bicinchoninic acid assay (CoWin Biosciences). An equivalent amount of protein was separated by 10% sodium dodecyl sulfate-polyacrylamide gel electrophoresis and transferred to polyvinylidene fluoride membranes (Millipore, Burlington, MA, USA). Then, the membranes were blocked with 5% bovine serum albumin (Solarbio) and subsequently probed using respective primary antibodies overnight at 4 °C. Membranes were then incubated with the secondary antibody conjugated with horseradish peroxidase (ZSGB-BIO, Beijing, China) for two hours at room temperature. Finally, the target proteins were measured by enhanced chemiluminescence (Thermo Fisher Scientific, Waltham, MA, USA) and Chemi Doc™ XRS+ System (Bio-Rad Laboratories). Glyceraldehyde 3-phosphate dehydrogenase (GADPH) (AB-P-R-001#, 1:1000 dilution, GOODHERE, Hangzhou, Chnia) served as the internal control. The information of primary antibodies were listed as followed: anti-LPCAT1 (ab214034, 1:1000 dilution, Abcam, Cambridge, MA, USA), anti-EGFR (A-10, 1:1000 dilution, Santa Cruz, CA, USA), anti-p-EGFR(F-3,1:1000 dilution, Santa Cruz, CA, USA), anti-PI3K (ab40755, 1:1000 dilution, Abcam, Cambridge, MA, USA), p-AKT (S473) (D9E, 1:1,000, CST, MA, USA), t-AKT(C67E7, 1:1,000, CST, MA, USA), and horseradish peroxidase (HRP) conjugated anti-rabbit secondary antibody (ab205718, 1:5000 dilution, Abcam, Cambridge, MA, USA).

### *In vivo* xenograft assay and ethics statement

PC-9R cells were prepared by suspending 1 × 10^6^ cells in 200 μL of serum-free PBS, then subcutaneously inoculating them into the right rear flanks of five-week-old female BALB/c nude mice. Gefitinib was prepared by dissolving a 250-mg clinical-grade tablet in 150 μL of sterile water of 0.05% Tween-80 (Sigma-Aldrich, St. Louis, MO, USA). When the volume of the transplantation tumor reached 100 mm^3^ (volume = 0.5 × a × b^2^, where a is the long diameter and b is the short diameter), the mice were treated with different drugs as indicated below (n = 3 per group). Xenograft tumor volumes were measured every three days when they were palpable.

Mice were treated with (1) gefitinib (10 mg/kg) once daily via oral gavage, (2) LY294002 (2.5 mg/kg) every three days via intraperitoneal injection, (3) gefitinib and LY294002 co-treatment at the same doses, or (4) an equal volume of diluents as control therapy. The mice were humanely euthanized using conventional cervical dislocation after 12 days of treatment. Tumors were excised and dissected for further pathological evaluation. Hematoxylin and eosin staining was performed to evaluate the pathological and morphological changes in the xenograft tumor tissues. Ethical approval of this study was obtained from the Ethics Committee of the Jiujiang University Affiliated Hospital.

### Statistical analysis

The data are presented as mean ± standard deviation values. Intergroup differences between two groups were assessed by the Student's t-test. Differences among multiple groups were assessed by one-way analysis of variance. The comparisons between the groups after the analysis of variance test were confirmed with the Student-Newman-Keuls method. In the present study, the experiments conducted were performed independently at least in triplicate. Significant differences were defined as when *p* < 0.05.

## Results

### LPCAT1 expression in PC-9R and PC-9 cell lines

**Figure 1:**
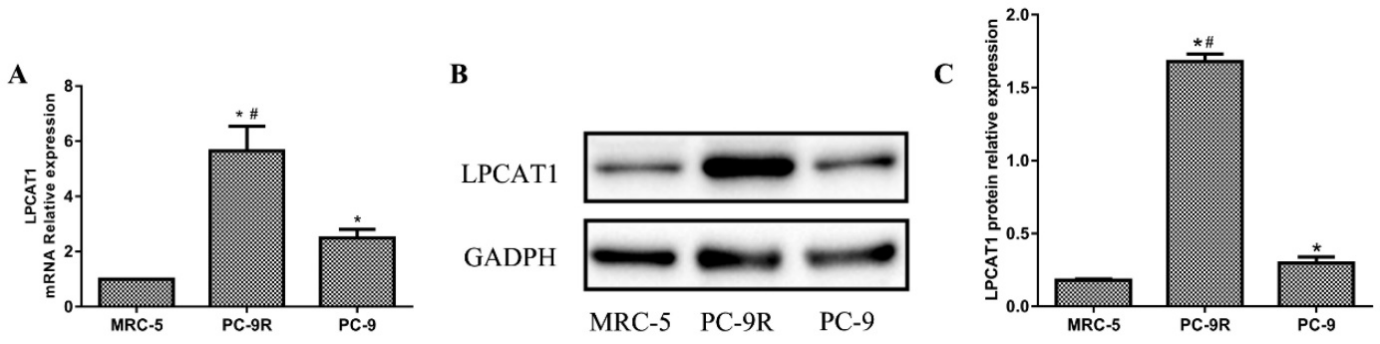
** The expression of LPCAT1 in PC-9R and PC-9 cell lines. (A)** The ranking order of LPCAT1 mRNA expressions from high to low expression was as follows: PC-9R, PC-9, and MRC-5 cell lines. **(B)** The protein expression level of LPCAT1 was validated by western blotting as the same order. **(C)** Bands were analyzed via densitometry using the Quality-One software (Bio-Rad Laboratories) (*p < 0.05 vs. MRC-5 group;**^ #^**p < 0.01 vs. PC-9 group).

Normal human embryonic lung cells (MRC-5 cells) were used as the control cell line. As shown in **Figure 1**, the mRNA expression of LPCAT1 in the PC-9R cell line was significantly higher than that in the PC-9 and MRC-5 cell lines. The PC-9 cell line displayed an enhanced LPCAT1 mRNA level relative to that of the MRC-5 cell line (**Figure 1A**). Similarly, the ranking order of LPCAT1 protein levels in descending order of expression was as follows: PC-9R, PC-9, and MRC-5 cell lines (**Figures 1B and 1C**).

### Sensitivity to gefitinib in PC-9 and PC-9R cell lines

**Figure 2:**
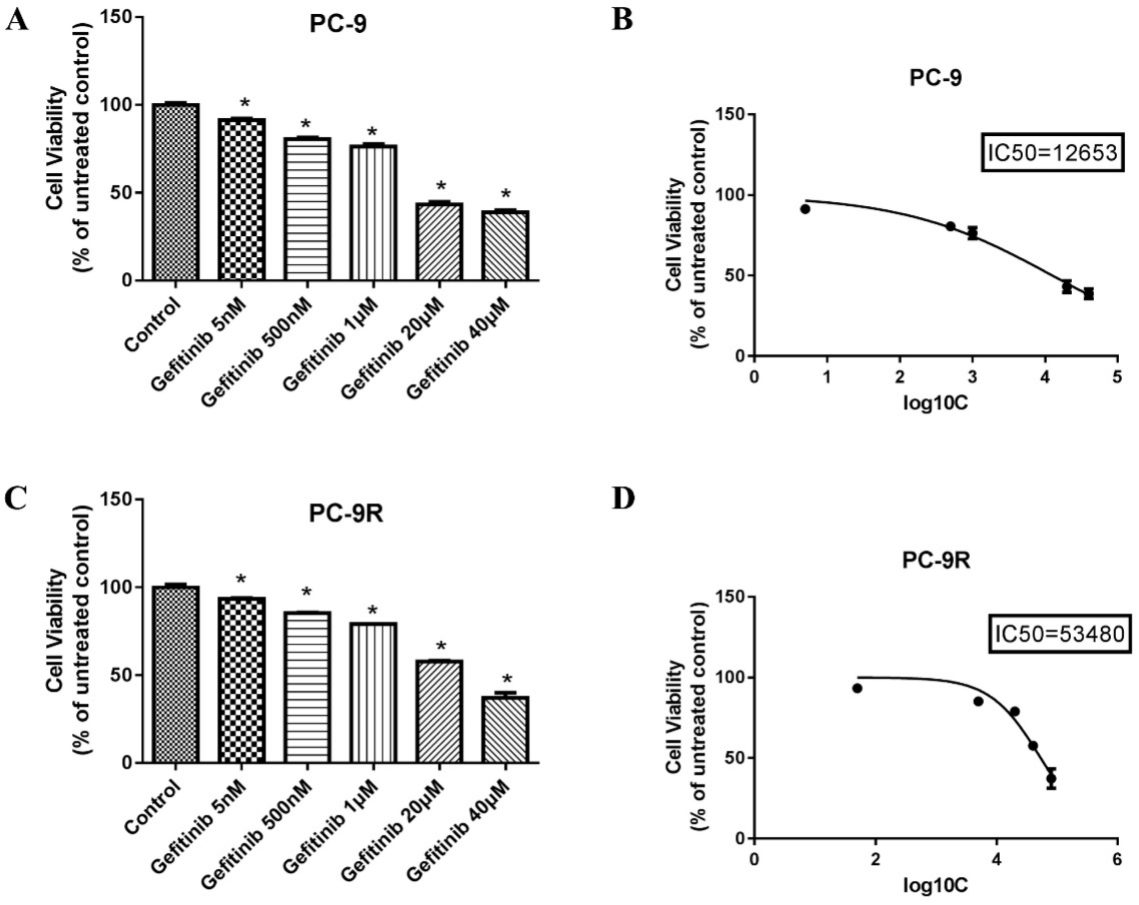
** Sensitivity to gefitinib in PC-9R and PC-9 cell lines. (A and B)** PC-9 cell lines were treated with different concentrations of gefitinib for 48 hours. The IC_50_ of PC-9 cells was 12,653 nM. **(C and D)** PC-9R cell lines were exposed to various concentrations of gefitinib for 48 hours. The IC_50_ of PC-9R was 53,480 nM. **(B, D)** The abscissa represents the log10C and C represents concentration; then, the ordinate represents the percentage (*p < 0.05 vs. control group).

To explore the sensitivity to gefitinib in PC-9 and PC-9R cell lines, we conducted a cell viability analysis by CCK-8 method. As evidenced in **Figures 2B** and** 2D**, the IC_50_ values of gefitinib in the PC-9 and PC-9R cell lines were 12,653 nM (12.653 μM) and 53,480 nM (53.48 μM), respectively, which further verified the resistance of PC-9R cells to gefitinib relative to that of PC-9 cells. Considering experimental efficiency, we chose the concentrations of 12.653 μM and 53.48 μM as the optimal ones for the following experiments.

### Validation of LPCAT1 transfection by qRT-PCR and western blotting

**Figure 3:**
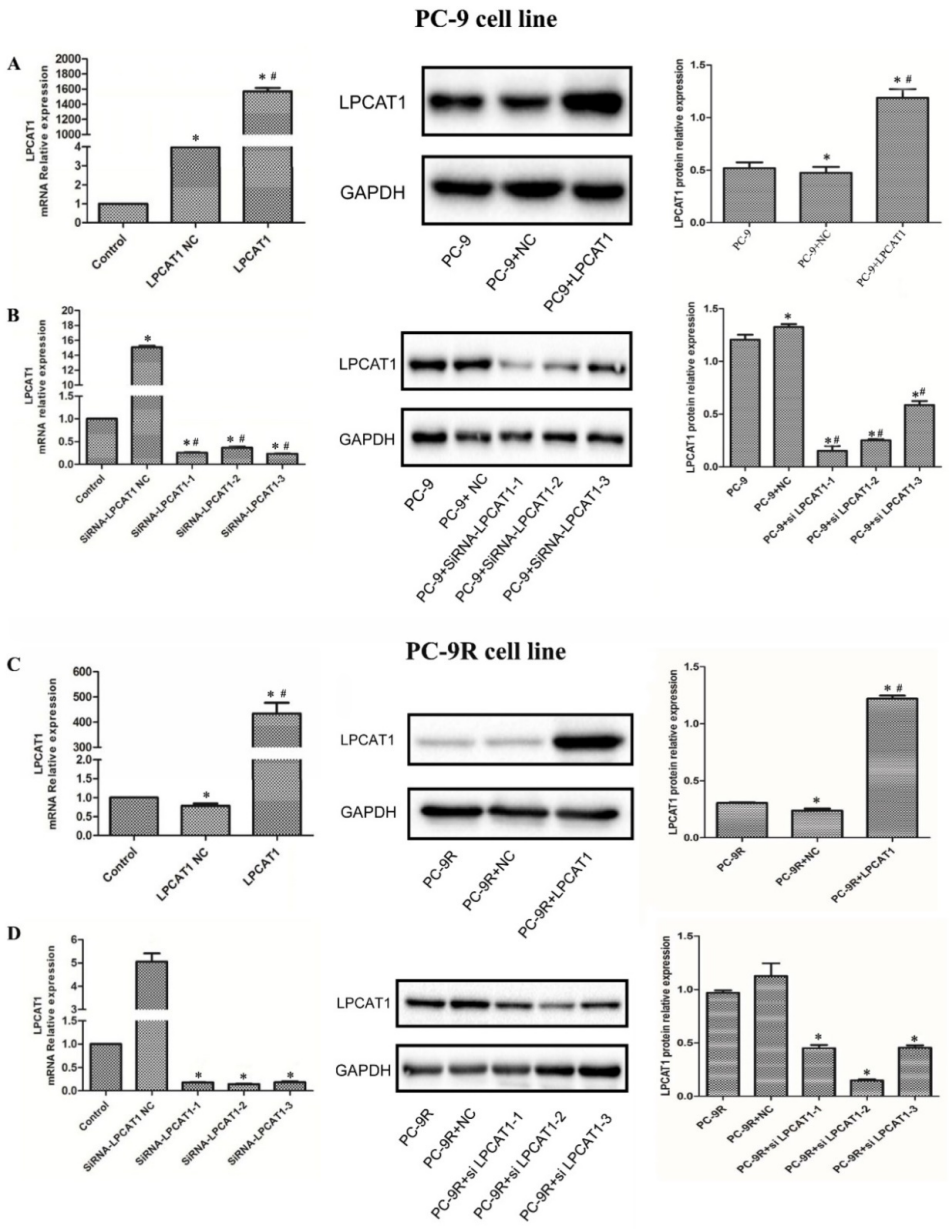
** Validation of LPCAT1 transfection via qRT-PCR and western blotting. (A)** The PC-9 cell line was transfected with LPCAT1 overexpression. **(B)** The PC-9 cell line was transfected with LPCAT1 siRNA to knock down LPCAT1. **(C)** The PC-9R cell line was transfected with LPCAT1 overexpression. **(D)** The PC-9R cell line was transfected with LPCAT1 siRNA to knock down LPCAT1. The expression levels of LPCAT1 mRNA and protein were examined by qRT-PCR and western blotting. [*p < 0.05 vs. control (PC-9 or PC-9R); **^#^** p < 0.01 vs. PC-9 NC or PC-9R NC].

In this study, we first transfected PC-9 and PC-9R cell lines with the LPCAT1 overexpression vector and empty vector, respectively. As presented in **Figure 3**, the levels of LPCAT1 mRNA and protein were robustly elevated in groups treated with the LPCAT1 overexpression vector compared to in groups treated with the empty vector and NC among either PC-9 or PC-9R cell lines by qRT-PCR and western blotting (**Figures 3A and 3C**). Furthermore, we designed three siRNAs to interfere with LPCAT1 expression in PC-9 and PC-9R cell lines and, as shown in **Figure 3**, either the PC-9 or PC-9R cell line in siRNAs groups exhibited lower levels of LPCAT1 mRNA and protein than did the empty vector and NC groups. These findings substantiated that the transfection was successful.

Owing to the highest interference efficiency being present in the PC-9R cell line, we chose siRNA2-LPCAT1 for the subsequent experiments (**Figures 3B and 3D**) (and hereafter refer to this as the “interfere” group).

### Effect of LPCAT1 knockout on cell proliferation and apoptosis in PC-9 and PC-9R cell lines

In the previous section, the LPCAT1 level of PC-9R cells was markedly higher than that of PC-9 cells. Thus, we further explored the effects of LPCAT1 on cell proliferation and apoptosis in the PC-9 and PC-9R cell lines. According to previously mentioned methods in this study, LPCAT1 was successfully knocked down via siRNA in either the PC-9 or PC-9R cell line, i.e., interference treatment.

**Figure 4:**
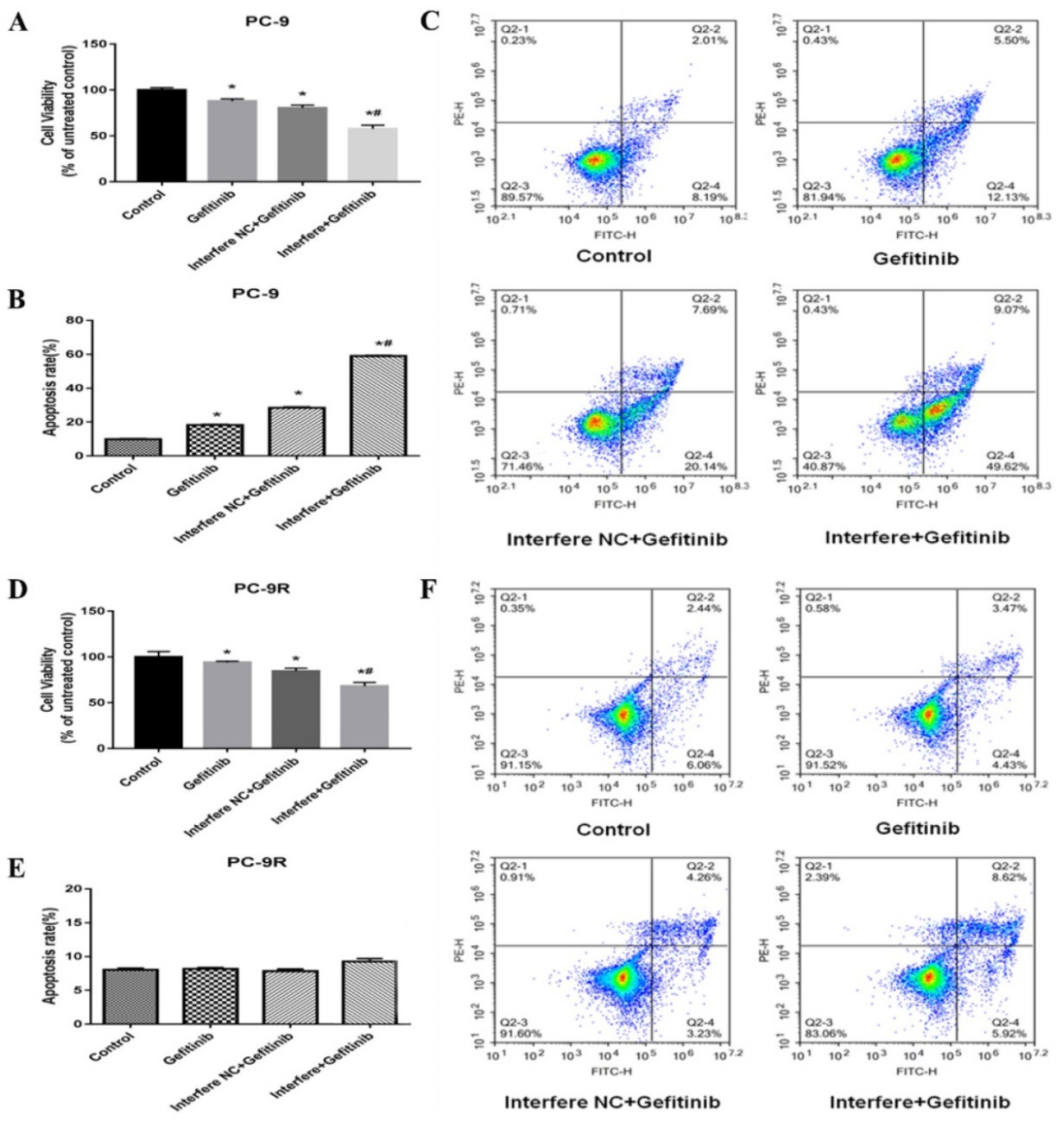
**Effect of LPCAT1 knockout on cell viability in PC-9 and PC-9R cell lines**. **(A)** Cell proliferation of PC-9 cells was analyzed by CCK-8 assay. **(B and C)** Cell apoptosis of PC-9 cells is shown using flow cytometry graphs. **(D)** Cell proliferation of PC-9 cells was examined by CCK-8 assay. **(E and F)** Cell apoptosis of PC-9R cells was detected by flow cytometry (*p < 0.05 vs. control group; **^#^** p < 0.01 vs. gefitinib group).

As illustrated in **Figure 4**, cell viability was markedly reduced in the gefitinib/interfere group relative to in the gefitinib/interfere NC group, gefitinib group, and control group, of either PC-9 or PC-9R cells (**Figures 4A and 4D**), which strongly indicated that LPCAT1 knockdown restored gefitinib sensitivity and inhibited cell proliferation in the PC-9R cell line.

In the PC-9 cell line, the cell apoptosis rate was significantly higher in the gefitinib/interfere group compared to in the other three groups (**Figures 4B and 4C**). However, the interference of LPCAT1 siRNA failed to induce cell apoptosis in PC-9R cell lines. These results suggest that other mechanisms (e.g., arresting the cell cycle) are involved in the proliferative inhibition of PC-9R cells transfected with LPCAT1 siRNA.

### Effect of LPCAT1 on the EGFR/PI3K/AKT pathway in the PC-9R cell line

In the present study, 25 μM of LY294002, an inhibitor of the PI3K/AKT pathway, was chosen as the experimental concentration according to the literature by Jiao *et al.* 15. To explore how LPCAT1 expression influences gefitinib resistance in LUAD, we employed LPCAT1 siRNA and overexpression vector to interfere and upregulate LPCAT1 and used LY294002 to inhibit the PI3K/AKT pathway in the PC-9R cell line, and luciferase siRNA served as the control group.

**Figure 5:**
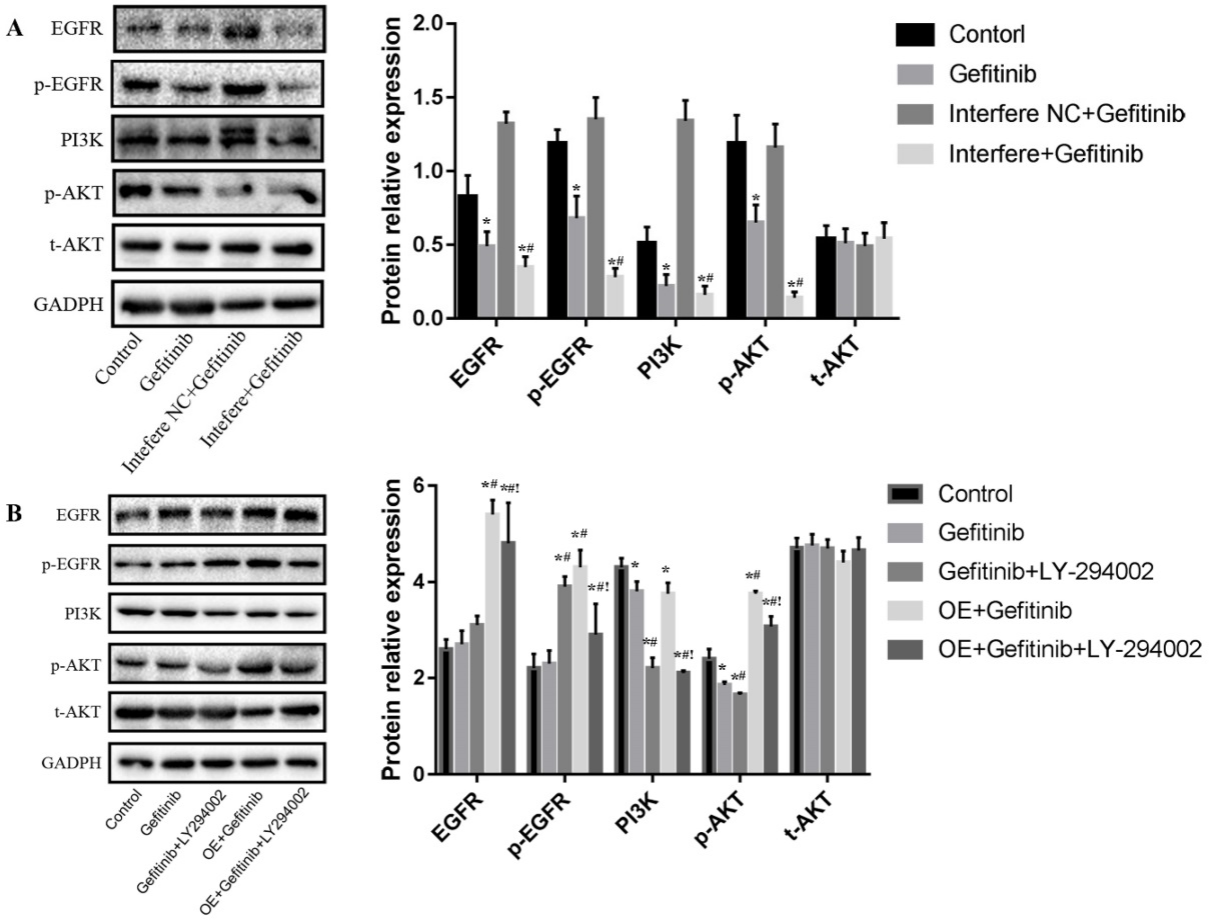
**Effect of LPCAT1 siRNA and overexpression on the EGFR/PI3K/AKT pathway in PC-9R cells. (A)** LPCAT1 siRNA was stably transfected into PC-9R cells. The cells received the treatments of vehicle (control) and/or gefitinib. **(B)** PC-9R cells were transfected with LPCAT1 overexpression vector. The cells were treated with vehicle (control), gefitinib, and/or LY294002. The expressions of EGFR, p-EGFR, PI3K, p-AKT, and t-AKT proteins were examined by western blotting. Interfere: LPCAT1 siRNA; interfere NC: LPCAT1 siRNA negative control; OE: overexpression of LPCAT1 (*p < 0.05 vs. control group; **^#^**p < 0.05 vs. gefitinib group;** !**p < 0.001 vs. gefitinib + LY294002 co-treatment group).

As shown in **Figure 5A**, gefitinib treatment markedly reduced the levels of EGFR, p-EGFR, PI3k, and p-AKT proteins relative to those in the control group among PC-9R cells. In comparison with in the gefitinib group, interfere LPCAT1 plus gefitinib treatment substantially reduced the expressions of EGFR, p-EGFR, PI3K, and p-AKT proteins in PC-9R cells. No significant change in the total-AKT (t-AKT) protein expression was observed. As depicted in **Figure 5B**, compared to in the gefitinib group, gefitinib and LY294002 co-treatment significantly decreased the levels of PI3K and p-AKT proteins. In comparison with gefitinib monotherapy, LPCAT1 overexpression distinctly elevated the expression levels of EGFR, p-EGFR, PI3K, and p-AKT proteins. Furthermore, the addition of LY294002 obviously decreased the levels of EGFR, p-EGFR, PI3K, and p-AKT proteins compared to the group of LPCAT1 overexpression plus gefitinib treatment among PC-9R cells. Similarly, there was no significant change in t-AKT expression. These findings above indicated that LPCAT1 was the important upstream molecule that regulated the EGFR/PI3K/AKT signaling pathway in the PC-9R cell line.

### Effect of LPCAT1 knockout on xenograft in PC-9R cell tumor-bearing mice

As the preceding results showed that LPCAT1-mediated gefitinib resistance of PC-9R cells is closely associated with the EGFR/PI3K/AKT pathway, we next verified this result by establishing a tumor-bearing mouse model.

**Figure 6:**
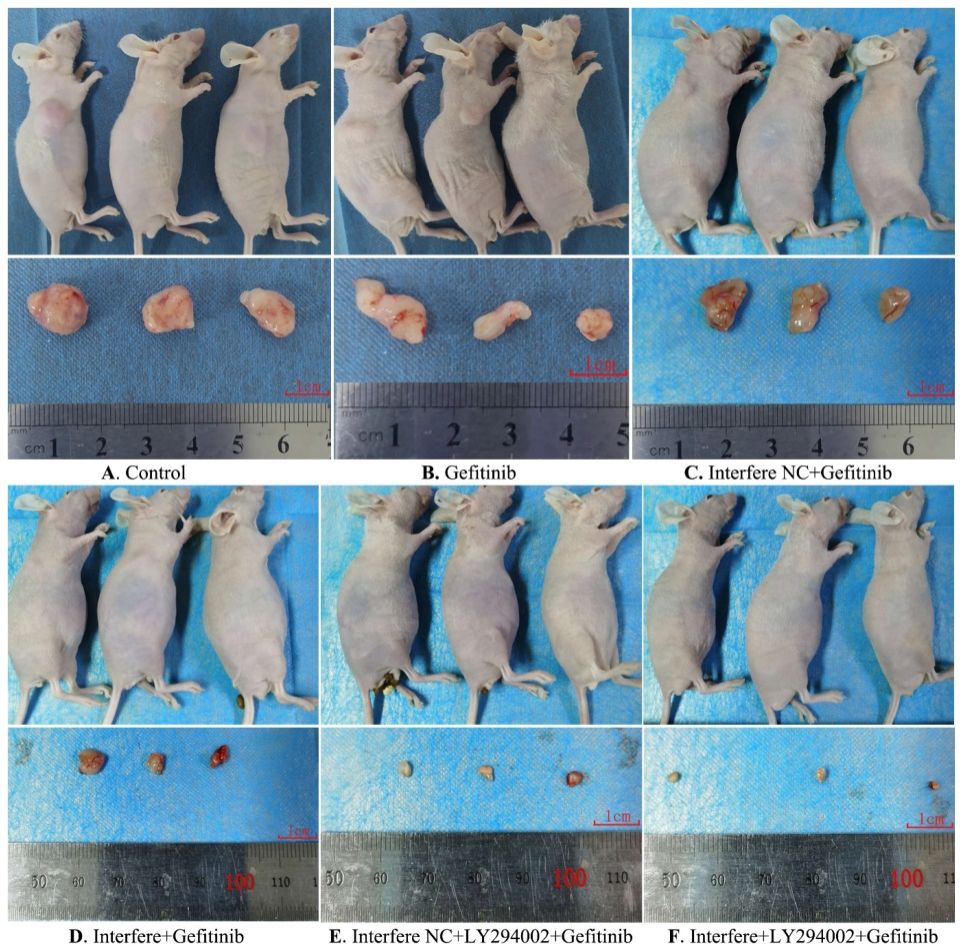
** Gross morphology of the xenograft tumor in different groups. (A)** Control group; **(B)** gefitinib group; **(C)** interfere NC; **(D)** interfere plus gefitinib; **(E)** interfere NC, LY294002 plus gefitinib; and **(F)** interfere, LY294002 plus gefitinib. Interfere: LPCAT1 siRNA; interfere NC: LPCAT1 siRNA negative control.

**Figure 7:**
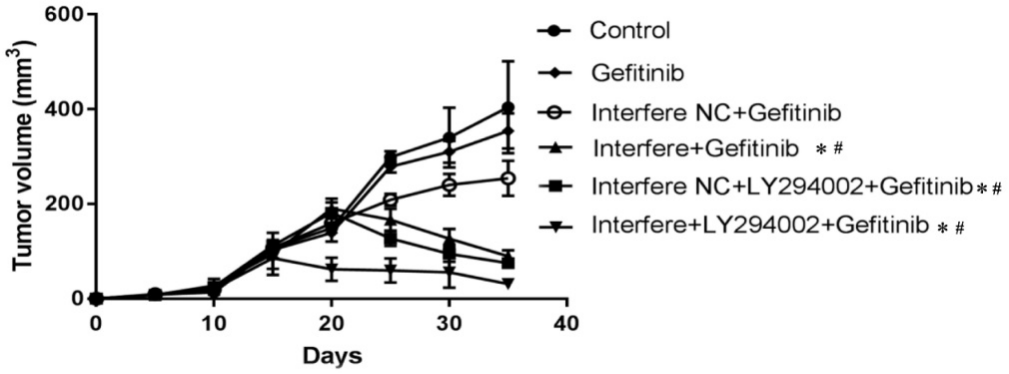
** The changes in xenograft tumor volume in different groups.** As the volume of the xenograft tumor reached 100 mm^3^, treatments were initiated with different drugs. The mice were humanely euthanized after 12 days of treatment. The changes in xenograft tumor volume were measured every three days. Graphs show mean ± standard deviation values (*p < 0.05 vs. control group; **^#^**p < 0.01 vs. gefitinib group).

As described in **Figures 6 and 7**, compared to in the control group, the volumes of xenograft tumors were slightly decreased in the groups of gefitinib and interfere NC plus gefitinib (**Figures 6A-6C and 7**). However, in comparison with gefitinib monotherapy, the treatment of gefitinib plus interfere group markedly diminished the volume of xenograft tumors (**Figures 6B, 6D, and 7**). Similarly, compared with interfere NC plus gefitinib, the addition of LY294002 substantially triggered tumor shrinkage (**Figures 6C, 6E, and 7**). In particular, the volume of the xenograft tumor dramatically shrunk in the combination group of interfere, LY294002, and gefitinib compared to that in the other five groups (**Figures 6A-6E and 7**).

**Figure 8:**
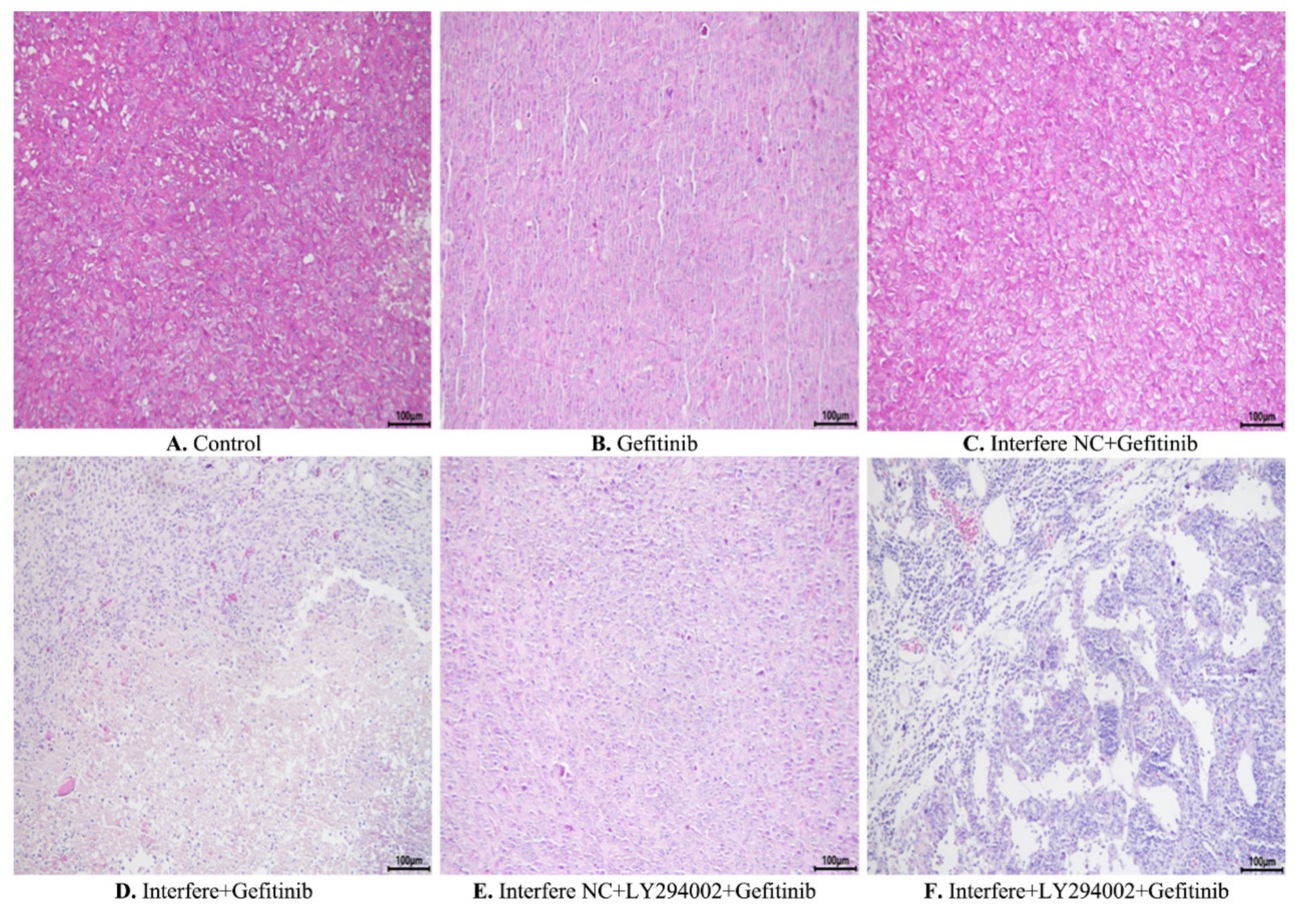
** Hematoxylin and eosin staining of xenograft tumors in different groups. (A)** Control group; **(B)** gefitinib group; **(C)** interfere NC; **(D)** interfere plus gefitinib; **(E)** interfere NC, LY294002 plus gefitinib; and **(F)** interfere, LY294002 plus gefitinib. Interfere: LPCAT1 siRNA; interfere NC: LPCAT1 siRNA negative control.

Furthermore, hematoxylin and eosin staining was conducted to elucidate the pathological and morphological changes in the xenograft tumor tissues. As illustrated in **Figure 8**, compared with the control group, gefitinib treatment led to slight cell necrosis and smaller cell nuclei (**Figures 8A and 8B**). In comparison with in the gefitinib group, cells in the groups of either interfere plus gefitinib or interfere NC, LY294002 plus gefitinib presented tumor cells with markedly smaller nuclei, greater cell necrosis, and larger gaps between cells **(Figures 8B, 8D-8E)**. Of note, the combination treatment of interfere, LY294002, and gefitinib resulted in massive tumor-cell necrosis, nuclei pyknosis, and lower intratumoral vascularity compared to the other five groups (**Figures 8A-8E**).

## Discussion

EGFR TKI, especially gefitinib, has widely been used in advanced NSCLC patients with EGFR-sensitive mutations and has dramatically improved the clinical outcome of NSCLC. However, nearly all NSCLC patients will eventually progress to an EGFR TKI-resistant status. The mechanism of EGFR TKI acquired resistance is very complex, including both T790M-positive and T790M-negative resistance, each of which account for about 50% of cases 4. For the latter, except for a few c-MET amplifications and RET rearrangements, the exact molecular mechanisms of EGFR TKI resistance have not been fully clarified.

In recent years, metabolic reprogramming, including lipid metabolisms, has been confirmed to contribute to tumor development and metastasis and drug resistance 16-18. Of note, LPCAT1, an important enzyme of phospholipid metabolism, has attracted much attention from oncology researchers and is involved in carcinogenesis and tumor progression 19. For instance, LPCAT1 overexpression was positively associated with cancer progression and could be a new biomarker in the prognosis of prostate cancer 20, 21. Also, LPCAT1 is overexpressed in triple-negative breast cancer, gastric cancer, and colorectal cancer, which correlates with poor prognosis and predicts early relapse 22, 23. In glioma cells, LPCAT1 induces the conversion of lysophosphatidylcholine from an unsaturated to a saturated state accompanying the remodeling of membrane lipids, resulting in the activation and recruitment of the EGFR pathway, which, in turn, further promotes the biosynthesis of LPCAT1. This has been referred to as the LPCAT1-EGFR positive feedback loop 19. The above findings indicated that LPCAT1 bridges the gap between the oncogene and metabolic reprogramming and consequently fuels tumor growth 24.

In normal lung tissue, LPCAT1 has been found to be highly expressed and to regulate surfactant lipid generation 25. Among patients with NSCLC, the prevalence of LPCAT1 was 80% and higher than that in normal lung tissues. Clinical analysis found that the median overall survival was significantly increased in NSCLC patients with high LPCAT1 expression levels compared to those with low LPCAT1 expression levels. Animal experiments demonstrated that LPCAT1 promoted brain metastasis of NSCLC *in vivo* 14. These results indicate that LPCAT1 participates in the tumorigenesis and progression of NSCLC. However, little is known about the role of LPCAT1 in resistance to EGFR TKIs in NSCLC cells.

A study *in vitro* revealed that LPCAT1 expression was markedly higher in the PC-9 cell line (EGFR 19 del E746_A750) than in the A459 cell line (EGFR wild-type, K-RAS mutation) and H460 cell line (EGFR wild-type) 14. This finding strongly suggests that LPCAT1 is closely correlated with EGFR gene mutations in NSCLC patients. In our study, we found that PC-9R cell line possessed substantially higher levels of LPCAT1 compared to its parental cell line, PC-9. Further analysis of cell viability indicated that LPCAT1 knockdown restored gefitinib sensitivity and inhibited cell proliferation in the PC-9R cell line. However, unlike the pro-apoptosis of LPCAT1 knockout in the PC-9 cell line, silencing LPCAT1 did not induce cell apoptosis in PC-9R cells. The underlying mechanism may be ascribed to the cell cycle arrest in the G1 phase induced by the suppression of LPCAT1 in PC-9R cells 14.

According to the foregoing literature, the LPCAT1-EGFR positive feedback loop contributes to the tumorigenesis of glioma cells 19. In cutaneous squamous cell carcinoma, LPCAT1 promotes progression via the EGFR-mediated AKT/p38MAPK signaling pathways 26. Coincidentally, EGFR/PI3K/AKT signaling has been closely associated with the occurrence of drug resistance and the transformation from LUAD to lung squamous carcinoma among NSCLC cells 5, 27, 28. Enlighted by these results, we investigated how LPCAT1 mediates resistance to gefitinib in the PC-9R cell line. In the present study, we found that knockout of LPCAT1 indeed significantly reduced the expression levels of EGFR, PI3K, and AKT proteins in the PC-9R cell line. Conversely, overexpression of LPCAT1 markedly enhanced the levels of EGFR, PI3K, and AKT proteins in the PC-9R cell line. Based on these findings, we can draw the conclusion that EGFR/PI3K/AKT cascade is the important downstream signaling pathway of LPCAT1 in LUAD. Finally, an *in vivo* study was performed to observe the effect of LPCAT1 knockout in PC-9R cell tumor-bearing mice. We found that, in the xenograft model, LPCAT1 silencing resulted in tumor shrinkage and restored gefitinib sensitivity in the PC-9R cell line. Further *in vivo* experiments demonstrated that LY294002 substantially circumvented resistance to gefitinib and promoted gefitinib-induced shrinkage of xenograft tumors on the basis of LPCAT1 knockdown. Of coincidence, the study by Zhou *et al.* reported that LY294002 exerted the activity of sensitizing EGFR wild-type NSCLC cell lines to erlotinib chemotherapy 29. These results strongly indicate that LPCAT1 can contribute to gefitinib resistance in LUAD by activating the EGFR/PI3K/AKT signaling pathway.

## Conclusions

In summary, our study reported that enhanced LPCAT1 was observed in the EGFR TKI-resistant cell line compared to the corresponding EGFR TKI-sensitive cell line. A functional investigation found that LPCAT1 contributed to the pathogenesis of gefitinib resistance in LUAD, the mechanism of which may be at least partly attributed to positive regulation of EGFR/PI3K/AKT signaling cascade. The present study also provided a novel insight into the mechanism underlying acquired resistance to EGFR TKIs from the perspective of phospholipid metabolism. Since high LPCAT1 expression exists in a variety of cancers, LPCAT1 may serve as a potential target of targeted therapy.

Of course, there are shortcomings in this study. One is that only a few single cell types were studied; the other is that the association of LPCAT1 with major resistant mutations (e.g., T790M mutation) was not explored. These questions will need to be further explored in our future research.
